# Retraction: Robust, highly active, and stable supported Co(ii) nanoparticles on magnetic cellulose nanofiber-functionalized for the multi-component reactions of piperidines and alcohol oxidation

**DOI:** 10.1039/d3ra90129g

**Published:** 2024-01-05

**Authors:** Pouya Ghamari Kargar, Ghodsieh Bagherzade

**Affiliations:** a Department of Chemistry, Faculty of Sciences, University of Birjand Birjand 97175-615 Iran P.Ghamari71@gmail.com gbagherzade@gmail.com bagherzade@birjand.ac.ir +98 56 32345192 +98 56 32345192

## Abstract

Retraction of ‘Robust, highly active, and stable supported Co(ii) nanoparticles on magnetic cellulose nanofiber-functionalized for the multi-component reactions of piperidines and alcohol oxidation’ by Pouya Ghamari Kargar *et al.*, *RSC Adv.*, 2021, **11**, 23192–23206, https://doi.org/10.1039/D1RA00208B.

The Royal Society of Chemistry hereby wholly retracts this *RSC Advances* article due to concerns with the reliability of the data.

The XRD pattern in Fig. 2a and the EDX data in Fig. 3a contain repeating sections.

The authors provided the raw XRD data for Fe_3_O_4_ in Fig. 2a of this article and the raw EDX data for Fe_3_O_4_ in Fig. 3a, but these were found to contain duplicated sections of data across different datasets representing different samples within this article and other articles.

The raw XRD data for Fe_3_O_4_ in Fig. 2a of this article was found to have a number of regions of data identical to the raw data provided by the authors for CuO in Fig. 4b of ref. 1 and Fig. 3 of ref. 2.

The raw EDX data for Fe_3_O_4_ in Fig. 3a of this article was found to have duplicated sections of data within the dataset and with the raw data for Fe_3_O_4_@NFC@NSalophCu)CO_2_H in Fig. 4c of ref. 2.

The authors have stated that they outsourced the XRD and EDX data collection to an external company.

Given the significance of these concerns, the findings presented in this paper are no longer reliable.

The authors were informed about the retraction of the article. Pouya Ghamari Kargar and Ghodsieh Bagherzade have not agreed with the decision.

Signed: Laura Fisher, Executive Editor, *RSC Advances*.

Date: 13th December 2023.

## Supplementary Material
